# Severe Intracranial Hemorrhage at Initial Presentation of Acute Myelogenous Leukemia

**DOI:** 10.5811/cpcem.2018.4.37881

**Published:** 2018-06-26

**Authors:** Amanda Balmages, Joseph Dinglasan, Megan Boysen Osborn

**Affiliations:** * Touro University California, Vallejo, California; † Mission Hospital, Department of Emergency Medicine, Mission Viejo, California; ‡ University of California Irvine Health, Department of Emergency Medicine, Orange, California

## Abstract

Intracranial hemorrhage (ICH) is the second leading cause of mortality among patients diagnosed with acute myelogenous leukemia (AML). The bone marrow failure associated with AML produces dysfunctional platelets, which significantly increases the risk of hemorrhagic complications within this population. In this report we discuss the case of a previously healthy female patient, newly diagnosed with AML, who rapidly developed fatal ICH.

## INTRODUCTION

Intracranial hemorrhage (ICH) is the second leading cause of mortality in patients with acute myelogenous leukemia (AML).^1^ AML-associated bone marrow failure produces marked thrombocytopenia with small, dysfunctional platelets, increasing risk of hemorrhage.^2^ Emergency physicians (EP) should maintain a high degree of suspicion for hemorrhagic complications in patients presenting with new-onset AML. Bleeding-type disseminated intravascular coagulation (DIC) is a common finding in AML patients, especially those with the acute promyelocytic leukemia (APL) subtype, and further increases risk of death from hemorrhage.^3^,^4^

DIC may be difficult to diagnose in the emergency department (ED) setting due to a lack of specific lab parameters and variable presentation, but a high degree of suspicion for DIC in AML patients is warranted. Administration of all-trans retinoic acid (ATRA) significantly improves outcomes of APL patients by preventing or rapidly reversing DIC.^5^ ATRA is a lifesaving intervention that EPs, in consultation with hematology-oncology, should consider in patients with laboratory values that suggest AML. Administration of platelet concentrate may also be useful.^2^ AML carries a relatively favorable prognosis if the complications of the disease – such as infection or hemorrhage – can be avoided or effectively managed.^6^ In this report we discuss a patient with newly diagnosed AML, who presented to the ED ambulating, alert, and oriented, and while en route to a higher level of care quickly deteriorated before further interventions could be implemented.

## CASE REPORT

A previously healthy 55-year-old female was evaluated in urgent care for easy bruising for three weeks’ duration. After she was found to have abnormal laboratory results, she was directed to a community ED for further treatment and care.

The patient presented to a community ED the following day. She denied trauma, fever, chills, headaches, or abdominal pain. Upon initial evaluation, the patient had a temperature of 98.5º F, pulse of 87/minute, respiratory rate of 18/minute, and blood pressure of 170/75 mm/Hg. Pulse oximetry showed 100% saturation on room air. Physical exam was unremarkable, except for ecchymosis to the upper and lower extremities bilaterally. Initial laboratory data was significant for a white blood cell (WBC) count of 51.7×10^9^/L, with 89% monocytes and 5% segmented neutrophils, platelets of 16×10^9^/L and hemoglobin of 11.3 g/dL. Prothrombin time (PT) was 17.3 seconds, and international normalized ratio (INR) was 1.6. Complete blood count was negative for blasts; however, Auer rods were present, and the specimen was sent for peripheral smear and flow cytometry. The EP consulted oncology by phone for suspicion of acute leukemia, and the patient was scheduled for an outpatient follow-up two days later, with instructions to return if her symptoms worsened.

Early on the day of her scheduled oncology consultation, the patient returned to the ED complaining of bilateral lower extremity pain and multiple new bruises. She had pain in her lower extremities, from thighs to feet, and occasionally buttocks. She denied tingling, numbness, bladder or bowel incontinence, back pain, or headache. Review of systems was positive only for gross hematuria. Other than mild tachycardia, vital signs at triage were within normal limits. Similar to the prior visit, her physical exam showed ecchymosis over all four extremities, but was otherwise unremarkable. Neurologic examination was within normal limits.

Hematological studies showed significant dysfunction of multiple cell lines, including WBCs of 110.8×10^9^/L, platelets of 60×10^9^/L, hemoglobin of 10 g/dL, 0% neutrophils, and blasts now at 22%. Additional labs found a PT of 18.7 seconds, INR of 1.6, and a d-dimer of 27.9 mg/L FEU. Initial analysis of peripheral smear showed multiple blast forms with convoluted nuclei and monocytoid features. Flow cytometry results from the previous visit were consistent with AML, while a lack of cluster of differentiation antigen 34 (CD34) and human leukocyte antigen – antigen D related (HLA-DR) was suggestive of APL. After ruling out deep vein thrombosis by lower extremity ultrasound, the patient was transferred by ambulance to a tertiary care center. At the recommendation of the receiving oncologist, 30mg of ATRA was administered prior to transfer to prevent DIC.

While en route to the tertiary care facility, the patient became acutely altered and lost consciousness. Upon arrival, her pulse was 68/minute, blood pressure was 231/96, respiratory rate was 15/minute, and oxygen saturation was 99% on 15L by non-rebreather mask. The patient’s Glasgow Coma Score was 1-1-1, her left pupil was fixed and dilated, and she was intubated for airway protection. She was given 50g mannitol. The right pupil became fixed and dilated shortly thereafter, and another 50g mannitol was administered.

Computed tomography (CT) demonstrated a 7.2 centimeter parenchymal hematoma with associated edema and a 10mm midline shift, causing leftward uncal herniation (Image). Trace subarachnoid hemorrhage was also noted. The patient was emergently evaluated by neurology and neurosurgery and was treated with 60ml (30ml × 2) 23.4% sodium chloride. Neurosurgery evaluated the patient’s CT and reported that mortality associated with ICH of this size was 72%, and that full recovery, if she survived at all, would be unlikely. Although there was no advanced directive in place, the patient’s family members agreed that the patient would not have wanted surgical intervention if her chances of significant recovery were unrealistic.

Over the next 24 hours, the patient’s diagnostic studies demonstrated continued derangement across multiple parameters, with blast forms increasing to as high as 83%, fibrinogen dropping to 77 mg/dL, and platelets paradoxically oscillating from 60×10^9^/L, to 13×10^9^/L, to 88×10^9^/L in a matter of hours. Aspartate aminotransferase was measured at 64 units/L and alanine aminotransferase at 61 units/L, while creatinine increased from 0.9 mg/dL at initial presentation to 1.3 mg/dL, and troponin I was measured at 7.70 ng/mL at its peak. The patient died within 24 hours of arrival at the tertiary care center.

**CPC-EM Capsule**

What do we already know about this clinical entity?

Intracranial hemorrhage is a leading cause of death in patients with acute myelogenous

leukemia (AML) and can occur as a result of disseminated intravascular coagulation or bone marrow failure.

What makes this presentation of disease reportable?

The patient’s rapid progression makes this a valuable example of how critical a role the

emergency physician can play in the care and management of patients with new-onset AML.

What is the major learning point?

This report highlights the importance of emergency physicians in gaining familiarity with medications uncommonly used in the emergency department.

How might this improve emergency medicine practice?

The authors’ hope is that this will improve emergency medical practice by highlighting

the emergent nature of the complications of AML.

## DISCUSSION

The incidence of AML in the United States is approximately 20,000 cases per year.^7^ While infection is still the leading cause of death in this population, approximately 10% of patients will die from DIC or bleeding diathesis at the time of initial presentation of AML, with ICH as the most common hemorrhagic complication.^8^–^11^ Risk of death from ICH in this population is linked to extent of coagulopathy and leukocytosis; those patients found to have WBCs >100×10^9^/L and INR >1.5 have significantly higher risk of death from ICH than those found with lower respective lab values, while thrombocytopenia additionally increases risk.^11^,^12^ Female gender has also been associated with an increased risk of death from ICH.^10^, ^11^ Therefore, it may be prudent for EPs to consider admission or observation for any patient meeting these high-risk criteria.

While prophylactic platelet transfusion has been well studied and widely used in AML patients undergoing chemotherapy,^13^,^14^ according to blood transfusion guidelines, prophylactic platelet transfusion is not routinely performed for patients newly presenting with evidence of AML whose platelet counts are greater than 10×10^9^/L.^2^, ^15^ In 2011, Psaila et al. conducted one of the first studies that looked specifically at platelet function in patients with AML as compared to platelet function in equally thrombocytopenic individuals with immune thrombocytopenic purpura (ITP) and determined that patients with AML have more dysfunctional platelets than those in patients with ITP.^2^ This raises the question of whether it may be beneficial for EPs to consider prophylactic platelet transfusion at a higher platelet count in patients recently diagnosed with AML, who have not yet undergone any form of treatment.

DIC is especially common in patients with the APL subtype of acute leukemia; however, the risk of hemorrhage exists with all types.^1^ Pathogenesis of DIC in patients with APL is mediated by increased blast cell production of tissue factor (TF) and cancer procoagulant (CP), and blast cell overexpression of the surface protein annexin II, which acts as a co-receptor for plasmin and tissue plasminogen activator, leading to extensive fibrinolysis.^16^ Combined with thrombocytopenia and dysfunctional platelets, these factors can cause a patient to rapidly progress to overt DIC. Unfortunately, there is no single, specific test that is diagnostic of DIC, so a combination of studies must be obtained if DIC is suspected.^3^ According to a 2014 study published by Wada et al., the findings of PT prolongation, d-dimer elevation, fibrinogen reduction, and platelet count reduction, is the most reliable combination of test results readily attainable in the ED to indicate the bleeding type of DIC associated with hematologic malignancies.^3^

Administration of ATRA is effective in rapidly reversing DIC in these patients as it functions to stop aberrant production of TF and CP and simultaneously induce differentiation of leukemic promyelocytes.^5^ Current recommendations support starting ATRA as soon as a diagnosis of APL is suspected, as increased incidence of early death has been linked to delays in ATRA administration by as few as one to two days.^17^ Therefore, EPs may consider initiating ATRA treatment upon initial presentation of a patient with suspected APL. Prompt initiation of ATRA, followed by concomitant chemotherapy has significantly reduced morbidity and mortality in this population, and APL is now considered one of the most treatable subtypes of acute leukemia with long- term remission rates of up to 80% in those who survive the presentation period.^6^,^18^

## CONCLUSION

Patients with AML may access the ED for care throughout the course of their disease; they may be diagnosed in the ED, or they may present with infection, chemotherapy-induced nausea and vomiting, or hemorrhage. Maintaining a high degree of suspicion for ICH and DIC is important in improving outcomes of these patients throughout the course of their treatment. Emergency physicians may consider a variety of treatment strategies for managing the complications of AML. Gaining familiarity with medications uncommonly used by emergency physicians, such as ATRA, may be beneficial in preventing disastrous outcomes.

Documented patient informed consent and/or Institutional Review Board approval has been obtained and filed for publication of this case report.

## Figures and Tables

**Image:**
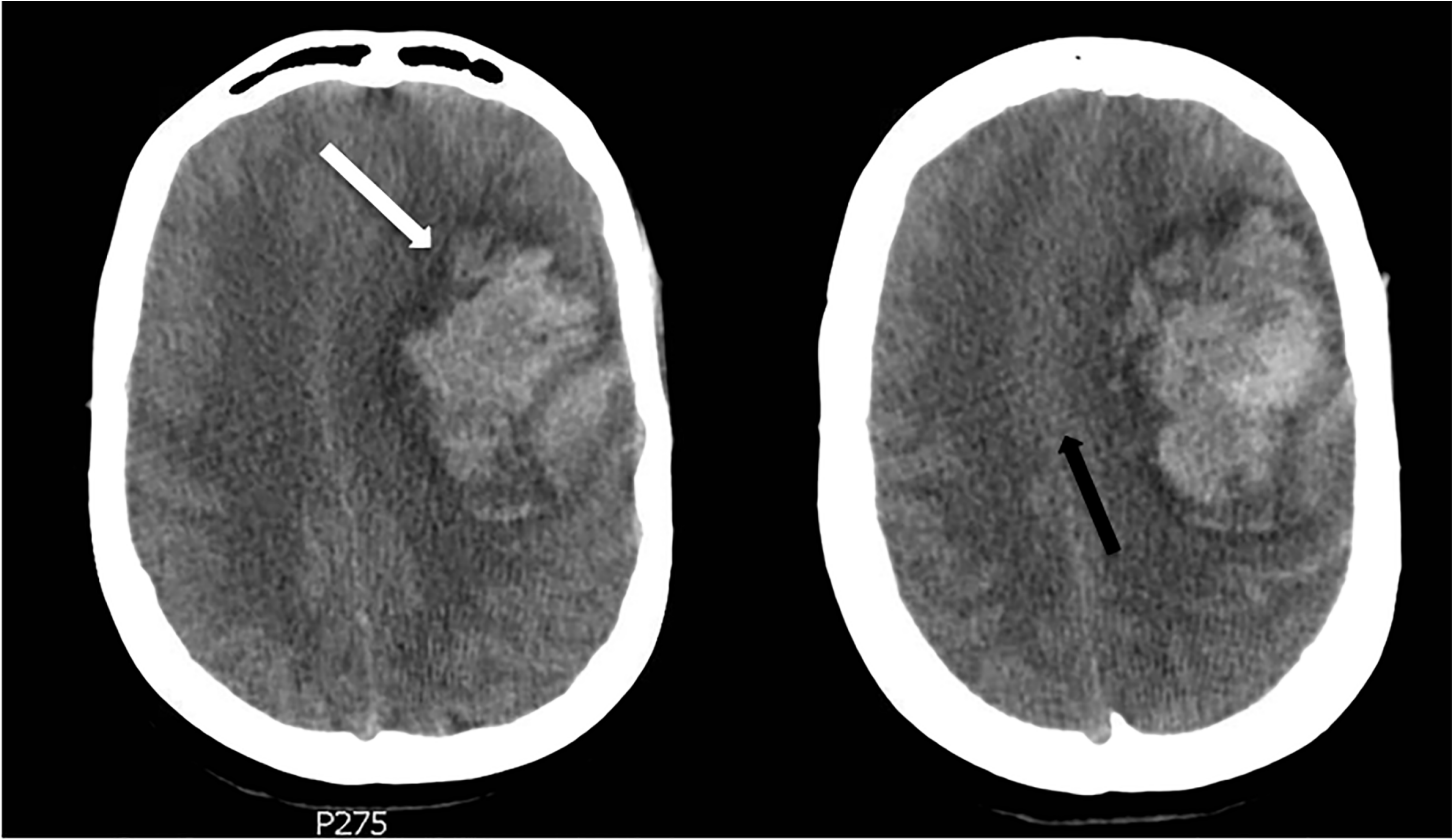
Computed tomography of the brain, axial images, demonstrating 7.2 centimeter parenchymal hematoma (white arrow) with associated edema causing 10 millimeters of midline shift (black arrow).

## References

[1] Chen C-Y, Tai C-H, Tsay W (2009). Prediction of fatal intracranial hemorrhage in patients with acute myeloid leukemia. Ann Oncol.

[2] Psaila B, Bussel JB, Frelinger AL (2011). Differences in platelet function in patients with acute myeloid leukaemia and myelodysplasia compared to equally thrombocytopenic patients with immune thrombocytopenia. J Thromb Haemost.

[3] Wada H, Matsumoto T, Yamashita Y (2014). Diagnosis and treatment of disseminated intravascular coagulation (DIC) according to four DIC guidelines. J Intensive Care.

[4] Miguel A, Sanz MA, Montesinos P (2010). Open issues on bleeding and thrombosis in acute promyelocytic leukemia. Thromb Res.

[5] Barbui T, Falanga A (2005). Hemorrhage and thrombosis in acute leukemia. Haematol Rep.

[6] Testa U, Lo-Coco F (2016). Prognostic factors in acute promyelocytic leukemia: strategies to define high-risk patients. Ann Hematol.

[7] Cancer Stat Facts: Leukemia - Acute Myeloid Leukemia (AML). Surveillance, Epidemiology, and End Results Program.

[8] Hersh EM, Bodey GP, Nies BA (1965). Causes of death in acute leukemia: A ten-year study of 414 patients from 1954–1963. J Am Med Assoc.

[9] Chang HY, Rodriguez V, Narboni G (1976). Causes of death in adults with acute leukemia. Medicine (Baltimore).

[10] Arbuthnot C, Wilde JT (2006). Haemostatic problems in acute promyelocytic leukaemia. Blood Rev.

[11] Kim H, Lee JH, Choi SJ (2006). Risk score model for fatal intracranial hemorrhage in acute leukemia. Leukemia.

[12] Kim H, Lee JH, Choi SJ (2004). Analysis of fatal intracranial hemorrhage in 792 acute leukemia patients. Haematologica.

[13] Rebulla P, Finazzi G, Marangoni F (1997). The threshold for prophylactic platelet transfusions in adults with acute myeloid leukemia. N Engl J Med.

[14] Blajchman MA, Slichter SJ, Heddle NM (2008). New strategies for the optimal use of platelet transfusions. Hematology.

[15] Kaufman RM, Djulbegovic B, Gernsheimer T (2015). Platelet transfusion: a clinical practice guideline from the AABB. Ann Int Med.

[16] Ikezoe T (2014). Pathogenesis of disseminated intravascular coagulation in patients with acute promyelocytic leukemia, and its treatment using recombinant human soluble thrombomodulin. Int J Hematol.

[17] Altman JK, Rademaker A, Cull E (2013). Administration of ATRA to newly diagnosed patients with acute promyelocytic leukemia is delayed contributing to early hemorrhagic death. Leuk Res.

[18] Tamai H, Yamanaka S, Yamaguchi H (2015). Effective management of acute promyelocytic leukemia with high risk of fatal intracranial hemorrhage. Biol Medicine.

